# The Arms Race Between Bacteria and Their Viruses: Update on Phage Countermeasures Against Bacterial Defence Systems

**DOI:** 10.1111/1751-7915.70462

**Published:** 2026-11-02

**Authors:** Harald Brüssow

**Affiliations:** ^1^ Department of Neuro‐Urology Balgrist University Hospital, University of Zurich Zurich Switzerland; ^2^ Spinal Cord Injury Center Balgrist University Hospital, University of Zurich Zurich Switzerland

## Abstract

In this editorial, I summarize recent progress on phage‐encoded anti‐defence against bacterial anti‐phage systems (APS). Computational approaches based on structural predictions of co‐folding bacterial and phage proteins identified many new PAD sponge proteins. New anti‐CRISPR‐Cas systems were characterized that destroy cas12a mRNA, inactivate Cas12a by acetylation, engage the seed region of the crRNA, or prevent Cas3 from cleaving DNA. The phage defence protein Gad1 forms an octamer web that wraps around the complex of the Gabija APS. Tad1 and 2 are phage sponge proteins that sequester signalling nucleotides of the Thoeris APS. Tad3 to 7 interact directly with the ThsB sensor and Tad8 with the ThsA effector of the Thoeris APS. Phage sponge proteins were also described inactivating the CBASS and Pycsar APS. Still other phages use phosphodiesterases that cleave cyclic nucleotide signals to inactivate APS. Many bacterial APS effectors are NADase, destroying the essential metabolite NAD^+^. Phages evolved enzymes that resynthesize NAD^+^ from its cleavage products or proteins directly inactivating the NADase. Many phages degrade the bacterial genome which creates signals activating the Metis or the Kongming APS. Phages counteracted these APS by suppressing pathways leading to the creation of particular mononucleotides degradation products detected by these APS. Several APS cleave specific tRNA, phages responded by mutating cleavage sites in tRNA (anti‐PARIS) or increasing tRNA expression to compensate for their destruction (anti‐Septu). Phages counteract toxin‐antitoxin APS by evolving their own antitoxin or by blocking the toxin protein. Researchers described cases where phages escaped detection by mutating phage proteins detected by bacterial sensors. Against the stringent response phages responded by destroying alarmones or inactivating enzymes producing these stress signals. The arms race between bacteria and phages can involve complicated layers of defence and anti‐defences and both bacteria and phages encode multiple defence systems.

## Graphical abstract

Bacteria evolved anti‐phage systems such as Gabija defence that degrade the intracellular phage genome. Phages evolved counter‐defences such as the Gad1 protein which surrounds and inhibits the Gabija defence complex allowing phage replication. Many cases of phage anti‐defences were recently described.

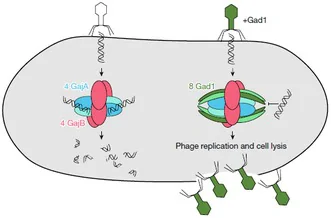

Bacteria and bacteriophages are locked in a continual conflict of infection and host defence. Recent research has revealed an increasing number of anti‐phage systems (APS) developed by bacteria to cope with the problem of predation by phages (for a recent review see Brüssow 2026). As this conflict is a matter of life or death for both bacteria and their viruses, the conflict resulted in an arms race (Hampton et al. 2020). Therefore, it should not surprise that researchers have described a likewise increasing number of viral strategies to evade these bacterial control systems (Mayo‐Muñoz et al. 2024). A review on phage anti‐defence systems (ADS) from 2024 (Murtazalieva et al. 2024) compiled 145 such systems. Although this large number of phage countermeasures might seem impressive, the authors noted that these ADSs counteract just 27 of the 152 different then known bacterial defence families. They hypothesized that many more of ADS remain to be detected in phages. Research on bacteriophage strategies to counteract bacterial APS is indeed very active and many new observations were reported over the last 2 years, which are summarized in the present editorial review on phage anti‐defence strategies.

## 1 Computational Approaches to New ADS

Instead of depending on serendipity discoveries of new ADS from studying individual phage‐host relationships, researchers explored a combination of computational and experimental high‐throughput approaches for their detection. Yirmiya et al. (2025) analysed 32 million unique phage proteins from a database and clustered them based on sequence similarity, followed by various filtering steps. Inclusion criteria for candidate phage proteins were lack of functional annotation, small in size and yield reliable folding predictions. This approach resulted in 38,000 protein clusters with at least 40 members. For a proof of principle demonstration, they have chosen on the bacterial side the ThsB protein (synthesizing a cyclic nucleotide signal) and the ThsA protein (NADase effector protein) from the Thoeris I APS. A representative from each of the 38,000 clusters was co‐folded with these two bacterial defence proteins in an iterative way. Fifteen phage proteins co‐folded with ThsB, four of them indeed inhibited ThsB (named Tad3 to 6 for Theoris anti‐defence) when engineered into a test phage blocked by the Thoeris I APS. Tad3 forms a homodimer that simultaneously binds two ThsB monomers. The scientists noted that the 11 remaining non‐inhibitory phage proteins showed only a small binding surface with ThsB. They therefore modified their prediction model by only including phage proteins displaying larger binding surfaces. When tested against Theoris II APS, two of the now three predicted candidate phage protein classes were associated with anti‐defence; both acted by inserting a loop into critical target ThsB protein sites. With this approach, they also identified a new anti‐defence protein active against two CBASS APS. The newly defined ADS were widely distributed among bacterial phyla. Notably, the phage‐encoded anti‐defence protein Tad4 bound and antagonized both a plant and a human immune protein, revealing ancestral relationships between defence and antidefense strategies shared among different kingdoms of life (Kranzusch 2026).

In a follow‐up study these researchers developed a prediction model for phage sponge proteins that bind nucleotide signals and thus block bacterial APS (Tal et al. 2026). The criteria used for sponge protein prediction were small proteins forming homo‐oligomeric assemblies that contained a deep, positively charged binding pocket for the nucleotide signal. This approach identified 120 candidate genes from the 38,000 protein clusters. The researchers constructed 62 plasmids, each expressing a pair of candidate genes, which were then tested in five bacterial strains expressing distinct APS, all based on cyclic nucleotide signalling. Nine phage proteins displayed anti‐defence properties. Four ‘Lockin’ proteins building cog‐like hexamers were identified that bound the signal molecules used in Thoeris I and II APS. By this approach also an Acb5 (anti‐CBASS) protein was identified that formed homo‐hexamers which cleaved the cGAMP signal molecule of the CBASS APS. More than 1000 homologues were found both for Lockin and Acb5 in phylogenetically diverse bacteria. The research group observed that phage sponge proteins can also occur as fusion proteins, consisting of two distinct domains (e.g., Tad2 and Acb4). Therefore, they looked for proteins that contained a Tad2 or an Acb4 domain combined with an unknown domain fold. They detected proteins that formed homo‐tetramers which sequestered the signalling nucleotide of the Thoeris I APS. Homologues of this ‘Sequestin A’ phage protein were found in hundreds of phages including T‐even phages.

## 2 Novel Anti‐CRISPR‐Cas Proteins

The 2024 review from Murtazalieva et al. (2024) mentioned 103 distinct families of anti‐CRISPR proteins (Acr) which are further classified as AcrI, AcrII, AcrIII, AcrV and AcrVI, named with Roman numbers according to the inhibited CRISPR‐Cas type. The mechanisms of action of Acr were manyfold and comprised inhibition of CRISPR‐Cas complex assembly, blocking the binding of target nucleic acids, prevention of the target nucleic acid cleavage, and degradation of cyclic oligonucleotide signalling molecules used by these anti‐phage systems (Figure S1). Take Cas12a as an example, a Type V CRISPR‐associated nuclease with broad utility for synthetic genome engineering, agricultural genomics, as well as biomedical and diagnostic applications. Inhibitors of Cas12a are thus of biotechnological interest. Previously, eight AcrVA proteins (AcrVA1 to AcrVA8) have been reported to target Type V CRISPR‐Cas systems and their molecular mechanisms have been described (Figure 1A). New mechanisms continue to be added as demonstrated by four recent publications.

**FIGURE 1:**
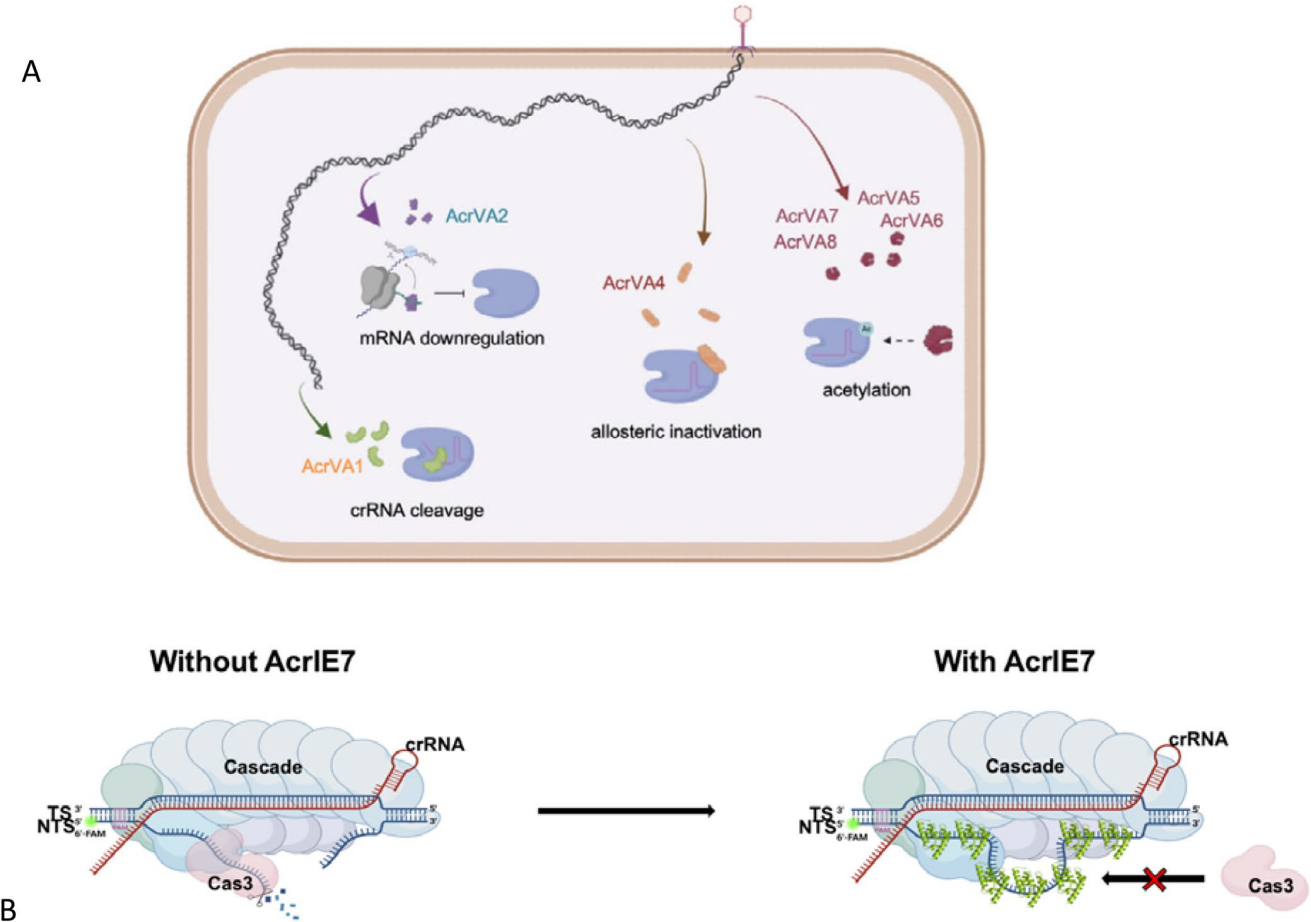
Newly discovered anti‐CRISPR‐Cas proteins. (A) Mechanisms of action for AcrVA proteins: AcrVA1 inhibits Cas12 through crRNA cleavage, AcrVA2 disrupts Cas12 biogenesis, AcrVA4 inhibits by allosteric binding, AcrVA5–8 inhibit via lysine acetylation. Figure credit: Han et al. (2026), open access under a Creative Commons Attribution License 4.0. (B) Model how AcrIE7 inhibits the type I‐E CRISPR‐Cas system that uses the multimeric Cascade complex to identify and degrade foreign DNA by nuclease‐helicase Cas3. AcrIE7 binds to the single‐stranded DNA in the R‐loop formed when Cascade encounters the target DNA (TS) that matches the CRISPR RNA (crRNA). AcrIE7 binding prevents Cas3 from cleaving the DNA. Figure credit: Figure from Kim et al. 2025, open access under a Creative Commons Attribution Licence 4.0.

### 2.1 AcrVA2

Transcriptomic analysis showed that *cas12a* was downregulated by AcrVA2 (Marino et al. 2026). The N‐terminal polypeptide region of Cas12a was necessary for AcrVA2‐mediated *cas12a* downregulation. Mutating a conserved four amino acid (aa) stretch in Cas12 abolished the AcrVA2 activity. AcrVA2 binds to the nascent polypeptide of Cas12a during translation and triggers immediate degradation of *cas12a* mRNA. AcrVA2 associates via its C‐terminal part with ribosomes and polysomes and triggers targeted co‐translational mRNA degradation upon binding the freshly synthesized N‐terminus of the Cas12a polypeptide. The 3′ end of the *cas12* mRNA that had not yet entered the ribosome is degraded. AcrVA2 does not itself cleave *cas12*, but recruits a cellular enzyme for *cas12* mRNA degradation. AcrVA2 is widespread among bacteria.

### 2.2 AcrVA5

In contrast, AcrVA5 inactivates the Type V CRISPR‐Cas system by acetylation of the Cas12a protein, which inhibited DNA cleavage. Mutating an aa in AcrVA5 important for acetyl‐CoA binding abolished the inhibitory activity of AcrVA5 (Duan et al. 2024).

### 2.3 AcrIIIA2

Subtype III‐A Csm is an RNA‐guided multi‐subunit CRISPR‐Cas immune complex that targets foreign RNA and DNA for degradation. It consists of five core proteins Csm1 (Cas10) to Csm5 and a crRNA guide. The system is activated when crRNA recognizes target RNA derived from plasmids or phages which triggers a cascade of reactions. This cascade includes target RNA cleavage by the Csm3 endonuclease, DNA degradation and the synthesis of cyclic oligoadenylates by Csm1 (Cas10), which in turn activates auxiliary ribonucleases, including Csm6. Johnson et al. (2025) demonstrated complex formation between the Csm1‐5‐effector and AcrIIIA2, an anti‐CRISPR encoded by a *Streptococcus thermophilus* phage. AcrIIIA2 needed an unusual chaperone for binding to the Csm1‐5 complex, namely the bacterial glycolytic enzyme enolase. Cryo‐electron microscopy (cryo‐EM) showed that the enolase‐AcrIIIA2 complex disrupts target phage RNA recognition by directly engaging the 3′ seed region of the crRNA, thereby preventing all downstream Type III immune responses. Here, AcrIIIA2 exploits a well‐conserved host factor known for its moonlighting activities to block CRISPR immunity.

### 2.4 AcrIE7

Type I CRISPR‐Cas3 consists of a Cascade multiprotein complex (Cas5, 6, 7, 8, 11) and an associated crRNA. Cascade glides along the DNA until it finds the target sequence corresponding to crRNA. After recognition of the target sequence, Cascade opens the DNA double strand, the crRNA forms an R‐loop (DNA–RNA hybrid) with one DNA strand. Cascade then recruits the Cas3 nuclease which degrades the uninvolved ssDNA strand. AcrIE7 is encoded by a *Pseudomonas* phage. AcrIE7 binds to the single‐stranded DNA in the R‐loop, and prevents Cas3 from cleaving the DNA (Kim et al. 2025) (Figure 1B).

## 3 Anti‐Gabija APS Phage Proteins Gad1 and 2

Yirmiya et al. (2024) exploited their collection of closely related *Bacillus subtilis* phages that were either resistant or susceptible to a widespread bacterial resistance system such as Gabija, which is found in 15% of bacteria. When aligning the phage genomes, they screened for genes that were found in all resistant phages but in none of the phages sensitive to Gabija APS. One such gene, *gad1* (Gabija anti‐defence 1), when engineered into a sensitive phage, conferred resistance to the Gabija APS. Homologues of this nearly 300 aa‐long Gad1 protein were found in nearly 100 phages from Proteobacteria and Firmicutes. Gabija purified in the presence of Gad1 was unable to cleave phage DNA. By the same approach, they identified a second phage gene, *gad2*, that encodes a nucleotidyltransferase which counteracts Gabija APS by an independent mechanism. Antine et al. (2024) elucidated the mode of action for Gad1 (Figure 2). The bacterial Gabija defence operons encode the proteins GajA and GajB. GajA contains a topoisomerase‐primase (Toprim) domain that catalyzes dsDNA breaks. GajB displays a helicase domain associated with ATP hydrolysis and nucleic acid unwinding. Both proteins are essential for phage defence in vivo. The GajA tetramer forms the core of the defence complex to which GajB dimers dock at opposite sides. The phage defence protein Gad1 forms an octamer web that wraps around the GajAB complex. This topological need explains why Gad1 is a large protein while most phage anti‐defence proteins tend to be small proteins. Gad1 prevented GajAB from binding to target DNA and abolished all nuclease activity in vitro.

**FIGURE 2:**
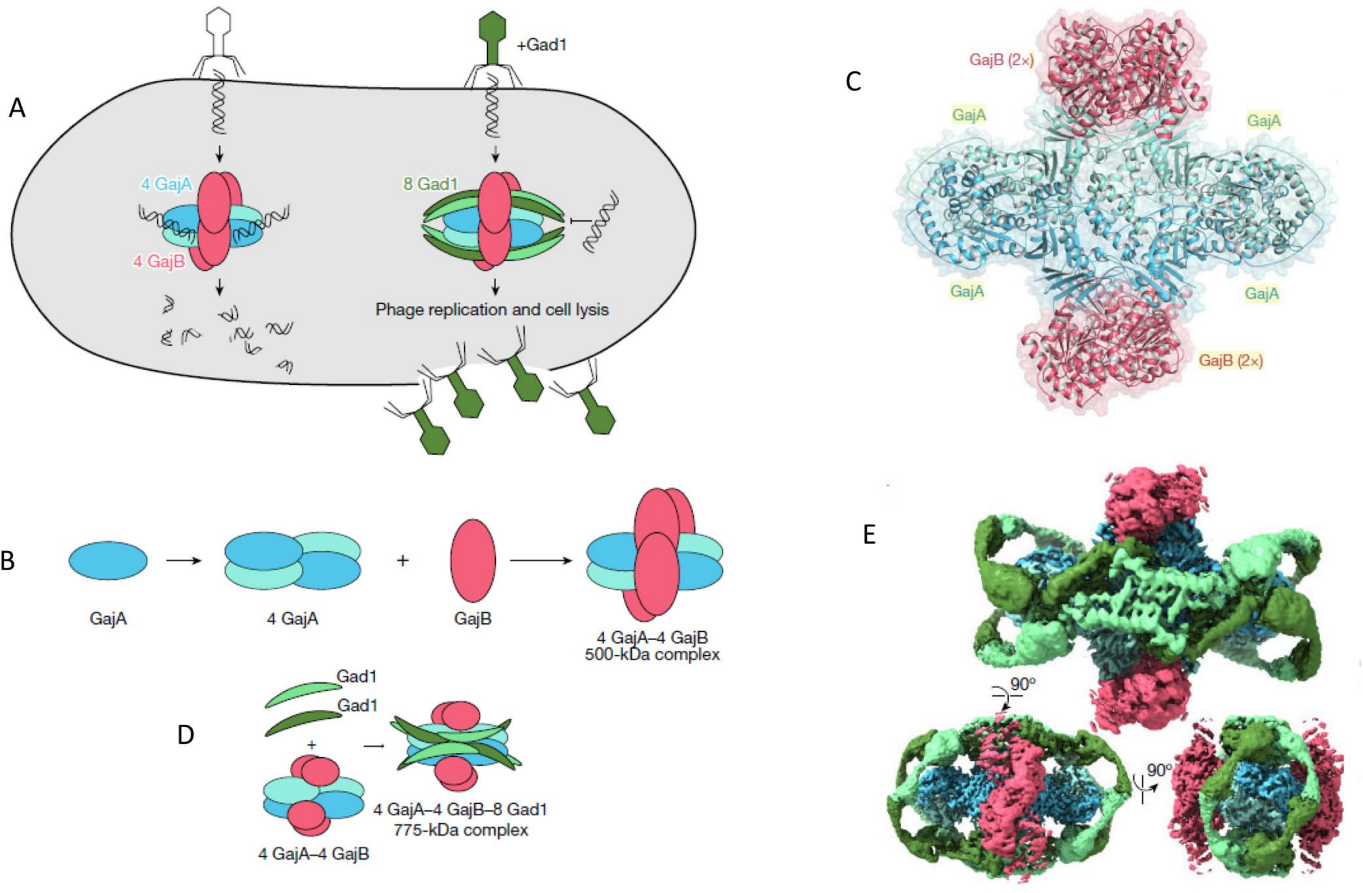
Viral evasion from the Gabija defence system. (A) The Gabija anti‐phage defense proteins GajA and GajB form a complex that degrades the injected phage DNA. The phage expressed Gad1 protein wraps around the GajA‐GajB complex, inhibiting its anti‐phage activity and allowing phage replication to continue. (B) The formation of the GajAB complex by GajA tetramerization and GajB docking. (C) Overview of the GajAB X‐ray crystal structure. GajA protomers are depicted in two shades of blue and GajB protomers are in red. (D) Schematic model of GajAB‐Gad1 co‐complex formation. (E) Cryo‐EM density map of GajAB in complex with Gad1, shown in three different orientations. The map is colored by the model in C, with Gad1 monomers depicted in two shades of green. Figure credit: Figure and legend from Antine et al. (2024), open access under a Creative Commons Attribution Licence 4.0.

## 4 Anti‐Hachiman APS Phage Protein Had1

The Hachiman APS consists of a two‐gene operon *hamAB* and has a broad‐spectrum anti‐phage function. HamA has DNA endonuclease activity, while HamB is a helicase. HamA binds with HamB to form the HamAB complex. This complex mediates DNA unwinding through ATP hydrolysis, followed by DNA degradation of both phage and host DNA (Cui et al. 2025). Yirmiya et al. (2024) described a small *Bacillus* phage protein Had1 (Hachiman anti‐defence) that overcame the HamAB APS. Had1 forms a homodimer with a central curved platform and splayed loops. Dimerization via hydrophobic packaging is essential for anti‐defence function. The center of Had1 contains a positively charged patch; the researchers suspected that it binds phage DNA and protects it from degradation by the HamAB complex.

## 5 Anti‐Thoeris Phage Proteins Tad

### 5.1 Tad1 and 2

The Thoeris APS consists of two proteins: a sensor ThsB that detects phage invasion and elaborates distinct nucleotide ADP‐ribose signals. The nucleotide signal binds and activates the effector protein ThsA which stops phage infection by different mechanisms. The molecular identity of the nucleotide derivates and that of the effector protein led to a definition of distinct types of Thoeris APS. Upon detection of phage infection, the TIR protein (containing a Toll/interleukin‐1 receptor) domain of ThsB processes nicotinamide adenine dinucleotide (NAD^+^) into ADP‐ribose (ADPR) derivatives as immune signals, which then activate downstream effectors, such as NAD^+^‐consuming enzymes (Type I Thoeris) (Figure S2 left) or a caspase‐like protease (Type IV Thoeris) (Figure 3B). As a countermeasure, phages developed sponge proteins that sequester the immune signals of Type I, II and IV Thoeris APS. Tad1 and Tad2 were discovered by again using comparative phage genomics approaches with *Bacillus* phages sensitive and resistant to Type I and II Thoeris APS (Yirmiya et al. 2024). Tad2 functions by binding and sequestering the ThsB produced signalling molecule gcADPR (Figure S2 right). The crystal structure of Tad2 revealed a homotetramer protein assembly which creates two identical binding pockets for the signalling molecule. Tad1 and Tad2, albeit molecularly different, both function as sponges for the gcADPR signal, preventing the activation of the NADase. However, Tad1 sequesters gcADPR through a structural fold completely different from Tad2. Tad1 also sequestered cyclic dinucleotides (CDN) and cyclic trinucleotides (CTN) used in CBASS and Pycsar APS, respectively. Tad 1 forms a hexamer that binds two CTNs in distinct pockets, separated from gcADPR‐binding sites. Point mutations abolished CTN but not gcADPR binding. In contrast, CDN and gcADPR bind to the same pocket in Tad1. The researchers showed that Tad1 antagonizes two CBASS APS including one that uses a phospholipase as effector protein, in addition to blocking Thoeris I function using an NADase as effector protein. Tad2 tetramer can simultaneously bind two CDN and two gcADPR molecules at distinct binding sites. Thoeris and CBASS are two APS that use different signalling molecules to mediate immunity. Tad 1 and Tad2 thus represent a unique class of anti‐defence proteins that inhibit more than one defence system (Li et al. 2024).

**FIGURE 3:**
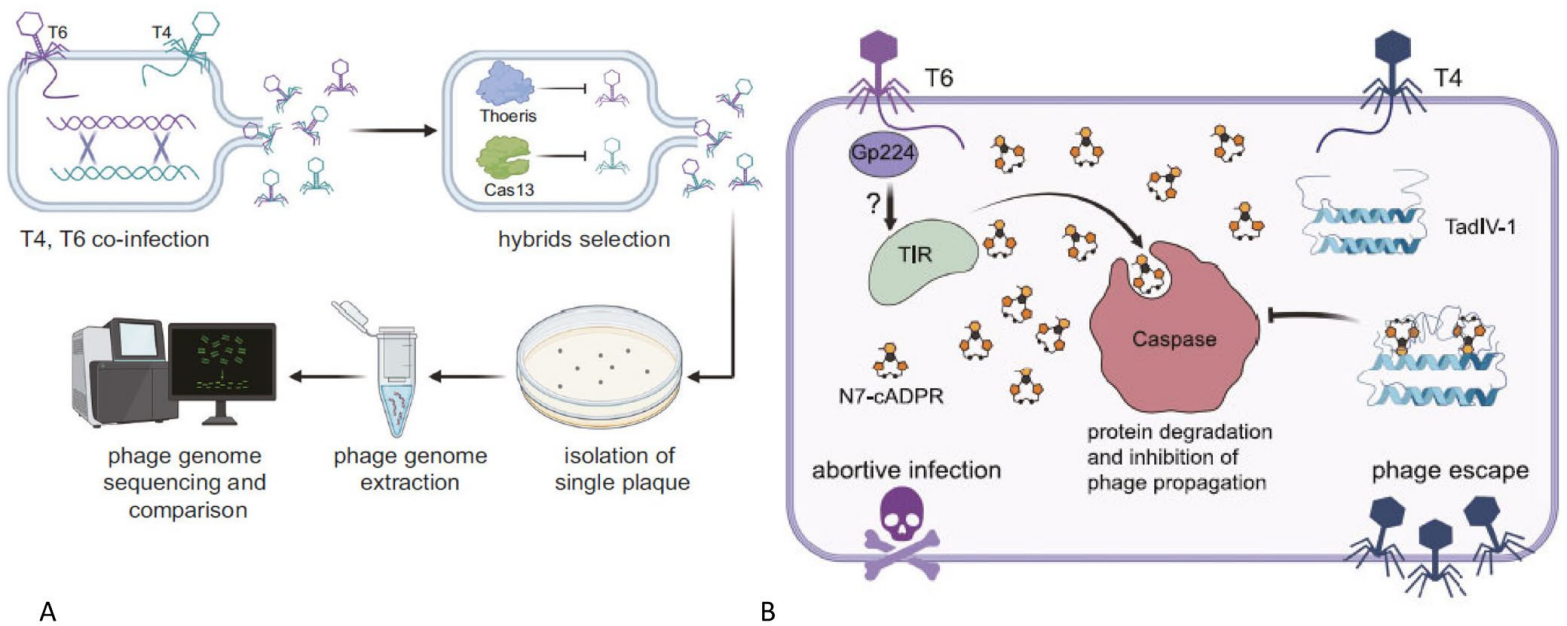
Illustration how the phage TadIV‐1 protein inhibits Type IV Thoeris APS. (A) Description of the strategy to identify TadIV‐1 protein via a phage mating assay. Replication of T4 and T6 in a sensitive strain allows genetic exchange of the two phages and generation of hybrids. Selection of the hybrids in the double defense strain (see text for details) resulted in the hybrids that are resistant to both defense systems. Isolation and sequencing of hybrid phages led to the identification of the TadIV‐1. (B) Mode of action for TadIV‐1. The phage protein functions as a sponge that sequesters the signal molecule and thereby inhibits Caspase effector activation which allows phage to escape from this APS. Figure credit: Figures from Chen et al. (2026), open access under a Creative Commons Attribution Licence 4.0.

### 5.2 TadIV‐1

The Type IV Thoeris APS generates the unique signal molecule N7‐cADPR to activate a Caspase‐like protease as effector. Chen et al. (2026) observed that coliphage T6, but not the closely related phage T4, was inhibited by the Type IV Thoeris APS. The researchers first coinfected a sensitive cell with both T6 and T4, allowing phage mating to occur, which created hybrid phages. They then plated the progeny phages on a cell construct that inhibited T6 (by Thoeris IV APS) and T4 (by CRISPR‐Cas13 using T4‐specific guide RNA). A few hybrid plaques were observed. They sequenced the phages and obtained uniformly hybrid phages where T6 phages had acquired the small T4 gene vs.*3* (Figure 3A). Deletion of vs.*3* from the hybrid phages abolished growth in Thoeris IV containing cells, while expression of Vs.3 in deletion mutants reconstituted phage multiplication. In biochemical experiments, Vs.3 sequestered the N7‐cADPR signal without degrading it. The sponge forms dimers in solution and the highly flexible N‐terminal end of the protein bound specifically the N7‐cADPR signal (Figure 3B). No binding of signalling molecules used by the Thoeris II, Kongming, or CBASS APS was observed. Isolated in vitro evolution of T6 phage in sensitive cells resulted in mutants insensitive to the Thoeris IV APS. Sequencing of escape phages identified mutations in the T6 capsid vertex protein, suggesting that this phage protein is a trigger of the Thoeris IV phage detection system.

### 5.3 Tad3 to 8

In contrast, Thoeris anti‐defence phage proteins Tad3, Tad4, Tad5, and Tad6 were not sponge proteins sequestering the signalling molecules. In a pulldown assay, all these Tad proteins co‐purified with ThsB. Engineering by a knock‐in of tad3 into a phage sensitive to Thoeris APS inhibition reestablished phage growth. A crystal structure of the ThsB‐Tad3 complex revealed that Tad3 forms a homodimer that simultaneously binds two ThsB monomers. The N‐terminal helices of Tad3 interlock with ThsB and form a hydrogen bond with the catalytic site of ThsB, explaining how the synthesis of the signal molecule by ThsB is inhibited. Mutating the loop residues in Tad4 and Tad6 that interlock ThsB and are predicted to block the catalytic site of ThsB rendered phages again susceptible to Thoeris APS inhibition. Likewise, Tad7 binds the ThsB protein of Type II Thoeris and inserts a loop into the active site pocket. In contrast, Tad8 is predicted to form a homodimer and insert loops into the ThsA effector. The Tad8 loop occupies the same space that would be otherwise occupied by the His‐ADPR immune signalling molecule blocking signal reception by the effector protein (Yirmiya et al. 2025).

## 6 Further Phage Sponge Proteins

### 6.1 Acb2

Cyclic GMP‐AMP synthase (cGAS) is an evolutionarily conserved enzyme that plays a pivotal role in innate immunity against eukaryotic viruses. In bacteria, cGAS‐like enzymes (CD‐NTases) synthesizing cyclic dinucleotide (CDN) and cyclic trinucleotide (CTD) are used in cyclic‐oligonucleotide‐based anti‐phage signalling system (CBASS) to activate diverse effectors (e.g., NADase, DNase). Phages encode anti‐CBASS (Acb) proteins to escape control. Acb1 degrades the cyclic nucleotide messengers (see below) while Acb2 serves as sponge protein which sequesters the signal without degrading it. Researchers investigating Acb2 observed that Acb2 binds different CDN as well as CTD with high affinity (Cao et al. 2024). The Acb2 hexamer binds two CTNs with two distinct binding pockets that are far from the three pockets for the CDN binding. One Acb2 hexamer can simultaneously bind two CTNs and three CDNs. Acb2 protects phage from Type III‐C (CTN‐producing) and Type II‐A (CDN‐producing) CBASS, using different interfaces, highlighting the versatility of the Acb2 protein as a broad‐spectrum inhibitor of CBASS APS.

### 6.2 Acb4

Chang et al. (2025) developed a biochemical approach to identify additional Acb proteins. They screened lysates from cells infected with 57 *Escherichia coli* or *B. subtilis* phages for binding of a specific radiolabeled CDN. The *B. subtilis* phage SPO1 synthesized a sponge protein Acb4 shortly after infection that bound the signal molecule while excluding nucleotide species used for non‐phage defence physiological signals of the host cell. Using an activity‐guided fractionation combined with a mass spectrometry approach, they identified a small sponge protein encoded in a putative phage anti‐defence island. Acb4 subverted CBASS immunity in vivo.

### 6.3 Anti‐Pycsar Sponges

Hadary et al. (2026) used another approach. They constructed phylogenetic trees for Tad1, Tad2 and Acb2 and selected 84 homologues that were cloned. Each cloned sponge homologue was introduced into cells expressing one of seven defence systems (CBASS, Pycsar, Thoeris I, II and IV) that rely on highly diverse nucleotide signalling molecules. Pycsar (pyrimidine cyclase system for antiphage resistance) is a widespread APS in prokaryotes in which a cyclase synthesizes cUMP or cCMP from UTP or CTP, respectively. The cUMP signal activates the PycTIR effector, an NADase that depletes cellular NAD^+^ levels, while cCMP activates the PycTM transmembrane effector. Both pathways lead to an abortive phage infection (Tal et al. 2021). The readout was cell growth after infection with phages sensitive to the resistance system. Active sponge proteins allowed phage growth detected by collapse of optical density reading. Notably, 65 of the 84 sponge protein homologues abolished the phage defence function. Several were active against two different classes of defence systems (Pycsar and Thoeris; CBASS and Thoeris), enabling phages to use a single small protein to counteract multiple types of bacterial APS.

## 7 Phosphodiesterases (PDE) Cleaving Cyclic Nucleotide Signalling Molecules

### 7.1 Acb1 and Apyc1

Cyclic nucleotides used by CBASS and Pycsar APS are stable in lysates of uninfected cells but are rapidly cleaved in lysates of cells infected with a number of diverse phages. Hobbs et al. (2022) used activity‐guided fractionation, comparative genomics and bioinformatic analysis followed by cloning experiments to associate *E. coli* phage T4 gene 57B with cleavage of CBASS cyclic nucleotides and *Bacillus* phage SBSphiJ gene 147 with degradation of Pycsar cyclic nucleotides (Figure 4A). They re‐named these phage anti‐defence proteins Acb1 and Apyc1, respectively. The crystal structure of Acb1 showed a compact fold with a U‐shaped binding pocket that can accommodate CDN and CTN signalling molecules used by CBASS. The scissile phosphodiester bond is located above the active catalytic site of Acb1. Apyc1 showed a different protein fold and a distinct cleavage chemistry (Figure 4B). The broad specificity of Acb1 allows evasion of diverse CBASS operons with a single phage gene. This is a necessity in view of the multitude of different nucleotide forms used in APS and the size restriction for phage genomes.

**FIGURE 4:**
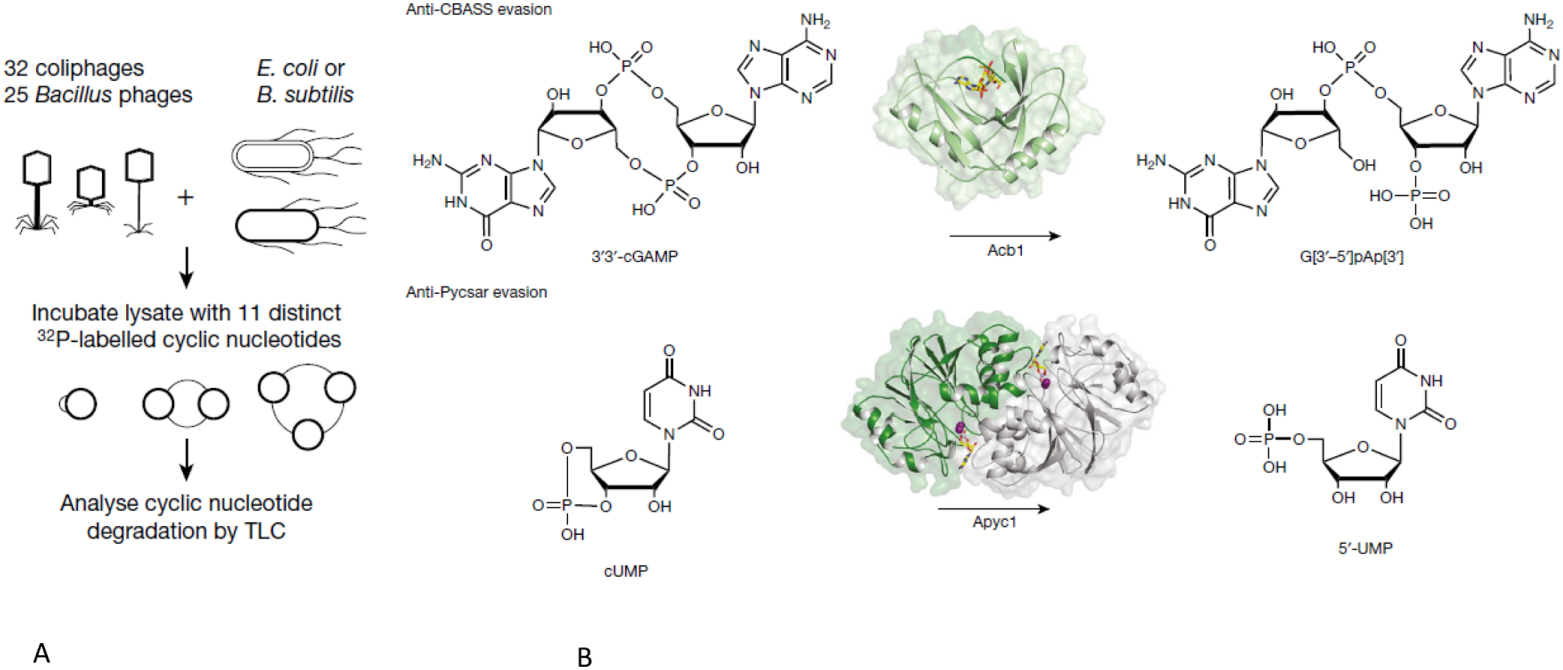
Phage phosphodiesterases cleave cyclic nucleotides produced by CBASS 1 and Pycsar 1 APS and thereby inactivate signal transmission in these APS.(A) The strategy used by Hobbs et al. (2022) to screen for cyclic nucleotide degradation activity in phage‐infected lysates using thin‐layer chromatography (TLC). (B) The cleavage reactions catalyzed by the phage Acb1 and Apyc1 phosphodiesterases with a structural model of the phage proteins above the reaction arrow. Figure credit: Figures from Hobbs et al. (2022), open access under a Creative Commons Attribution Licence 4.0.

### 7.2 Activity of a Poxvirus PDE in T4 Phage

Hobbs et al. (2024) reported that a poxvirus encodes a cGAMP PDE whose crystal structure showed a remarkable similarity with Abc1 up to the organization of the active site. The researchers demonstrated that the poxvirus enzyme and Acb1 degrade a wide and overlapping range of signalling nucleotides while some kingdom specificity was observed. When they deleted acb1 and acb2 from phage T4, the phage became susceptible to CBASS inhibition. Strikingly, when they engineered the poxvirus cGAMP PDE gene into this double deletion mutant of phage T4, phage growth was again observed in the presence of CBASS, demonstrating a functional cross‐kingdom interaction for evasion from host immunity.

Doherty, Nomburg, et al. (2025) used sequence similarity and structure alignments to define 75 PDE with catalytic histidine (‘2H PDE’) among viruses infecting bacteria and animals, defining 8 diverse enzyme groups. They analysed the signalling nucleotide cleavage specificity by thin layer chromatography. Acb1 showed the broadest cleavage pattern (Figure 4). This versatility of Acb1 allows escaping from many bacterial APS but also becomes a liability when degrading nucleotides involved in cellular regulation and in particular when degrading the signal molecule of the Panoptes APS that was evolutionarily designed as an inhibitory trap for phages using PDE for escape (Sullivan et al. 2025; Doherty, Adler, et al. 2025). Notably, a T4 phage chimera where Acb1 was replaced with a 2H PDE that does not cleave the Panoptes signal could grow in the presence of Panoptes APS but at the expense of a restricted evasion spectrum due to its more specific cleavage specificity.

## 8 Inactivating Antiviral NADase Functions

Not only the Type I Thoeris APS but numerous other APS (e.g., CBASS, DSR1, 2, SIR2–HerA) use as antiviral effector protein the NADase enzyme. NADase effectors cleave NAD^+^ into ADPR and nicotinamide. NAD^+^ is an essential cofactor for redox reactions used in oxidative phosphorylation, amino acid and fatty acid biosynthesis. NAD^+^ depletion interrupts cell metabolism and thus blocks phage replication since no cellular process can rebuild NAD^+^ from these cleavage products. This abortive infection mechanism is widespread in bacteria: 7% of bacteria encode NADases.

### 8.1 NARP and 2

As an anti‐defence countermeasure, 5% of sequenced phage genomes encode NAD^+^ reconstitution pathways. Osterman et al. (2024) elucidated two phage pathways achieving NAD^+^ reconstitution. NARP1 (NAD^+^ reconstitution pathway 1) (Figure 5 top) consists of two enzymes: Adps, an ADPR‐pyrophosphate synthetase, and Namat, a nicotinamide ADPR‐transferase. Co‐expression of these two proteins in bacteria expressing APS using NADase effectors abolished phage defence. Point mutations in the active sites of these two phage enzymes re‐established phage inhibition. The NARP1 operon was found in 4% of the sequenced phage genomes. NARP1 protected phages from the effects of NAD^+^ depletion independently of other phage factors and became active only when NADase was expressed. In vibriophages, a second NAD+ resynthesis pathway was identified: NARP2 (Figure 5 bottom). It consists of an enzyme synthesizing NMN from nicotinamide and PRPP and an enzyme forming NAD^+^ from NMN and ATP. However, NARP2 caused cells to produce excessive NAD^+^ even in the absence of NADase‐based APS. This situation would be a metabolic liability in the absence of NADase and might explain why NARP2 is only found in 1% of the sequenced phage genomes.

**FIGURE 5:**
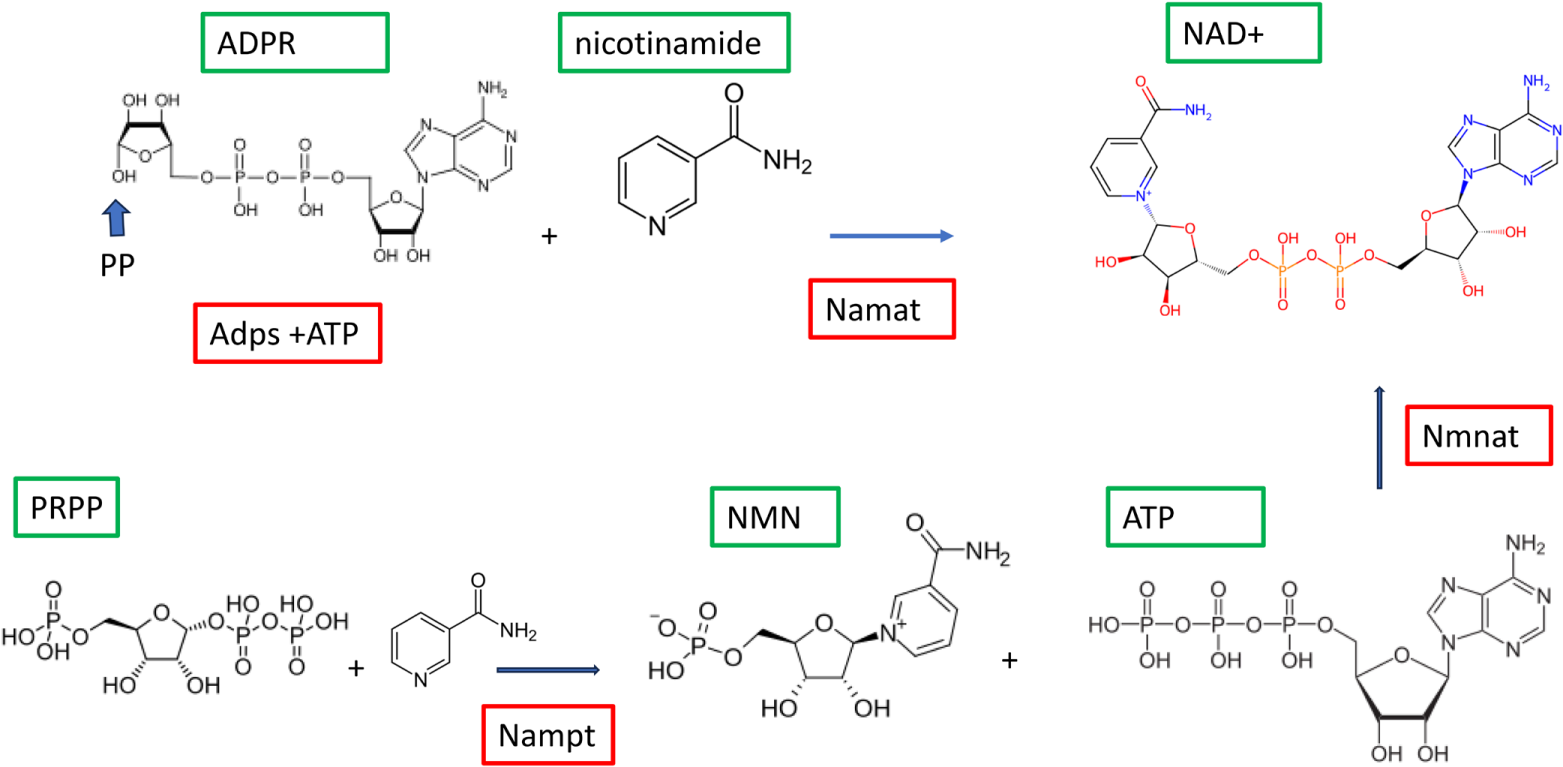
Illustration of two reaction pathways catalyzed by phage proteins that reconstitute NAD^+^ and thus counteract NADase activity expressed by diverse APS. Top: NAD^+^ reconstitution pathway 1 uses adenosine diphosphate ribose (ADPR) which is pyrophosphorylated by the phage enzyme Adps (ADPR‐pyrophosphate synthetase), and then coupled to nicotinamide by the phage enzyme Namat (nicotinamide ADPR‐transferase) to reconstitute NAD^+^ (Nicotinamide adenine dinucleotide). Bottom: The NAD+ reconstitution pathway 2 starts with PRPP (Phosphoribosyl pyrophosphate) which is coupled to nicotinamide by the phage enzyme Nampt (Nicotinamide phosphoribosyl transferase), the resulting NMN (Nicotinamide mononucleotide) is coupled to ATP by Nmnat (Nicotinamide mononucleotide adenylyltransferase) to give NAD^+^. Substrates are boxed in green, enzymes in red. Figure credit: Formula from Wikipedia in public domain.

### 8.2 Arms Race Around NADase

Osterman, Hurieva, Yirmiya, et al. (2026) described an additional layer of complexity in the arms race between bacteria and phages. They identified a standalone antiviral protein called aRES (the RES domain suggests an NADase, ‘a’ stands for anti, and it reads ARES following the convention to name antiviral systems after mythological characters, hence the sometimes peculiar name of such systems). aRES does the trick by cleaving NAD^+^ by a phosphorylase; the cleavage product is ADPR‐1P which the NARP1 pathway (in contrast to the NADP2 pathway) cannot use as a substrate. As NADP2 is metabolically wasteful due to its constitutive NAD^+^ synthesis, phages ‘found’ a simple trick to counteract aRES by adding a third gene to the NARP1 operon, namely a specific phosphatase transforming ADPR‐1P back to ADPR. Not enough with that, phages developed a second countermeasure to aRES in the form of a small protein aRad1 (aRES anti‐defence 1). aRad1 binds the aRES protein by penetrating the active site of aRES, thus blocking the NAD^+^ cleavage reaction. aRES senses phage infection by binding the phage T7 DNA polymerase, which activates its NADase function. Data like this underline the ingenuity of evolution developing tools in the arms race between phages and bacteria.

### 8.3 Nip

Still other phage anti‐defence strategies against NADase‐based APS were identified. The integrative element ICEBs1 from *B. subtilis* encodes the gene *spbK* which prevents propagation of the phage SPßP (Figure 6 top). SpbK binds the phage portal protein YonE which activates its NADase function that leads to an abortive infection. A closely related phage phi3T could multiply in the presence of SpbK. Loyo and Grossman (2025) associated the phi3T gene *nip* (for NADase inhibitor from phage) with escape from SpbK control. NIP bound to the NADase domain of SpbK, blocking its enzymatic activity although SpbK remained associated with YonE activator.

**FIGURE 6:**
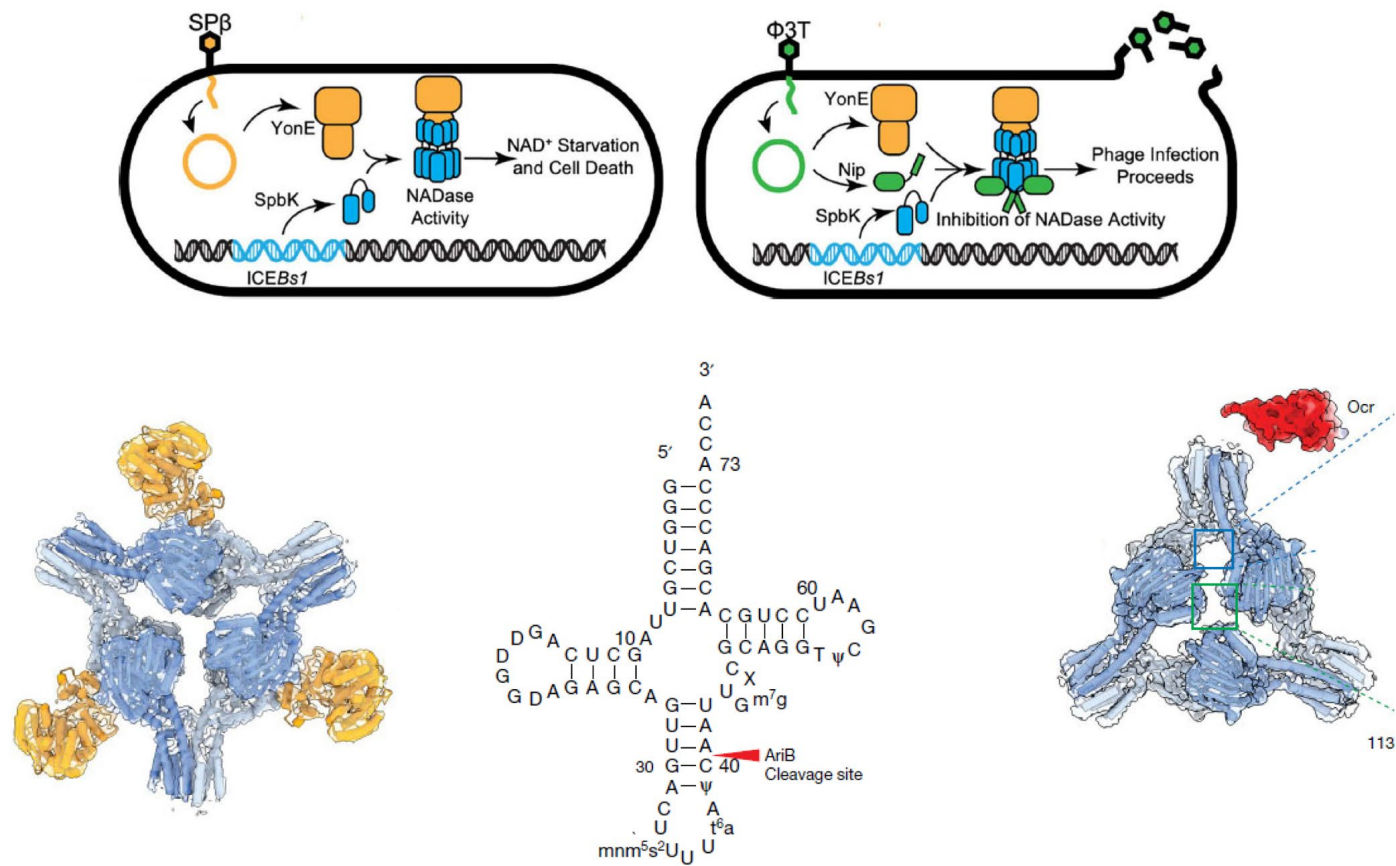
Mechanism of action for the phage 3T Nip protein inhibiting NADase activity and how phage T5 circumvents the Paris APS. Top: The integrative element ICEBs1 from *B* Φ3T Figure credit: Top‐ Figures from Loyo and Grossman 2025, open access under a Creative Commons Attribution Licence 4.0. NIP protein binds to the NADase domain of SpbK, blocking its enzymatic activity allowing phage replication. Bottom: The Paris APS consists of two proteins, AriA (blue) and AriB (yellow) that form a complex which is activated by the phage T7 protein Ocr (red). The released AriB cleaves lysine tRNA at the indicated site which leads to an arrest of protein translation. Phage T5 encodes a tRNA lacking this cleavage site. Figure credit: Bottom‐ Figures from Burman et al. 2024, open access under a Creative Commons Attribution Licence 4.0.

## 9 Countering Metis APS

Further bacterial APS are based on proteins with NADase activity. A recent addition is Metis consisting of a two gene operon *misAB* (Osterman, Hurieva, Moses, et al. 2026). MisA is a NADase which can also function as a standalone antiphage protein. MisA is activated by m^6^dAMP. As adenines are methylated only when incorporated in cellular DNA polymers, methylated mononucleotides signal infection with phages that degrade the host genome shortly after entry into the cell, as seen for phages T4, T5 and T7. Selecting escape phages that overcome the Metis APS yielded T4 phages mutated in *denA*, an endonuclease II enzyme responsible for the initial step of host genome degradation. These mutants did not degrade the host DNA genome but were still able to produce viable progeny.

## 10 Opposing Kongming APS

Kongming APS described by Zeng et al. (2025) consists of a three gene operon: KomABC. KomA deaminates dAMP to dIMP. A phage‐encoded kinase enzyme phosphorylates dIMP to dIDP which is further phosphorylated to dITP by a cellular enzyme. KomB serves as a phage sensor by detecting dITP. Binding of dITP to KomB leads to a conformational change in KomB which is necessary to activate the enzymatic activity in KomC which cleaves NAD^+^, depriving the cell of this essential cofactor for survival. Phage T5 is not susceptible to Kongming APS. Zeng et al. (2025) deciphered the resistance mechanism in phage T5. T5 degrades upon infection the bacterial DNA genome to dNMP. Instead of using them directly as precursors for phage DNA synthesis, T5 encodes a deoxyribonucleoside 5′‐monophosphatase (T5Dmp) degrading dNMP to dN. The removal of cellular dAMP, the precursor for dITP synthesis, prevents signalling to KomB. KomC is not activated and T5 can multiply in the presence of Kongming APS. The central role of T5Dmp in resistance to Kongming was demonstrated by deletion of this gene from T5 or substitution of a catalytic amino acid residue for the phosphatase activity of T5Dmp, which both rendered phage T5 again susceptible to Kongming APS.

## 11 Suppressing PARIS and Septu APS

PARIS defence system consists of two proteins, AriA and AriB (Burman et al. 2024). Ari A contains an ATPase domain, AriB a nuclease domain. AriAB forms a large complex of 430 kDa. The ATPase domains of AriA function as dimerization interfaces, creating a trimeric assembly of AriA homodimers that form the three blades of a propeller structure (Figure 6 bottom). AriB binds AriA between the propeller blades. The T7 phage anti‐restriction protein Ocr is the trigger for the PARIS APS. AriB dissociates from the complex in the presence of Ocr. AriB expressed alone does not provide phage defence; it needs a prior association with AriA. The released activated AriB forms a homodimer. Activation of the PARIS system leads to a rapid arrest of protein translation, followed by membrane permeabilization. The AriB dimer nuclease cleaves host lysine transfer RNA, causing translational arrest. T5 encodes a lysine tRNA that contains a mutation in the anticodon stem‐loop which resists AriB cleavage and allows the phage to multiply in the presence of the PARIS APS.

Further phage defence systems such as Septu and Retron Eco7 encode effector proteins (PtuA and PtuB) that lead to degradation of tRNA^Tyr^, resulting in an abortive infection. Azam et al. (2024) demonstrated that phages such as T1, T5, T7 harbour stretches of tRNA genes, including strongly expressed tRNA^Tyr^ genes that outcompete these APS. A distinct but parallel strategy to escape Septu inhibition was described for phage T6. Escape phages encode the phage homing endonuclease SegB that counteracts Septu by amplifying a genomic segment that contains the full‐length tRNA^Tyr^ gene. Amplification of the tRNA gene repeats increases the expression of the tRNA^Tyr^ gene, compensating for the tRNA degradation mediated by these APS (Chihara et al. 2026).

## 12 Inactivation of Toxin‐ Antitoxin Systems

Toxin‐antitoxin (TA) systems are widely distributed among bacteria but they have only recently been linked to phage defence functions (LeRoux et al. 2022). Earlier work had demonstrated that the DarTG system provides protection against several coliphages. Infection with T4 or T5 phages triggers release of the DarT toxins that can use NAD^+^ to ADP ribosylate DNA. ADP‐ribosylated phage DNA impairs both phage DNA and RNA synthesis. The DarG1 antitoxin is a NADAR superfamily ADP‐ribosylglycohydrolase that reverses these modifications in uninfected bacteria.

A T4‐like and a T5‐like phage escape DarTG control by mutating the phage DNA polymerase or via a phage protein that inhibits the DarT toxin. Johannesman et al. (2025) discovered an antitoxin in T‐even phages that counteracts the DarTG APS by removing ADP‐ribose modifications from phage DNA. The phage antitoxin is phylogenetically related to the bacterial DarG antitoxin protein (except for a loss of an N‐terminal domain), suggesting that it might have been acquired horizontally from bacteria.

DarTG systems are widespread in bacteria and have also been identified in the pathogen *Vibrio cholerae* where it inhibits the replication of the lytic vibriophage ICP1. Researchers identified a mutant ICP1 protein, Gp145, that blocked DarT toxicity as efficiently as DarG (Patel and Seed 2024). DarG antitoxin acts through two mechanisms: either as an enzyme that reverses the action of DarT (de‐ADP‐ribosylation) or by occluding the active site of DarT through mimicking negatively charged ssDNA. ICP1 Gp145 had no enzymatic activity but bound DarT. *V. cholerae* contains other APS active against vibriophage ICP1 such as a DNA nickase, a nuclease that degrades ICP1 phage DNA in infected cells (OLD APS). A mutated ICP1 protein, Gp205, binds to this nuclease and inhibits its phage degradation activity (Patel and Seed 2025).

Toxin‐antitoxin systems come in many forms. Type III TA consists of an endoribonuclease (RNase) toxin (ToxN) and antitoxin RNA (*toxI*), expressed as an array of short tandem repeats. The antitoxin RNA neutralizes the toxin RNase by sequestering it from other putative substrate RNAs (Figure 7B). During phage infection, the shut‐off of cell transcription leads to a loss of cellular RNA including *toxI*, hence ToxN is activated and degrades phage mRNA, blocking thereby the phage infection. Researchers evolved T4 to escape ToxNI APS. Escapers overcame ToxNI by segmental amplification of the T4 early gene *tifA*. TifA binds ToxN, occludes its active site and forms a high molecular complex with 16S rRNA. Phage mRNA thus escapes ToxN action and phage replication can proceed (Guegler et al. 2024).

**FIGURE 7:**
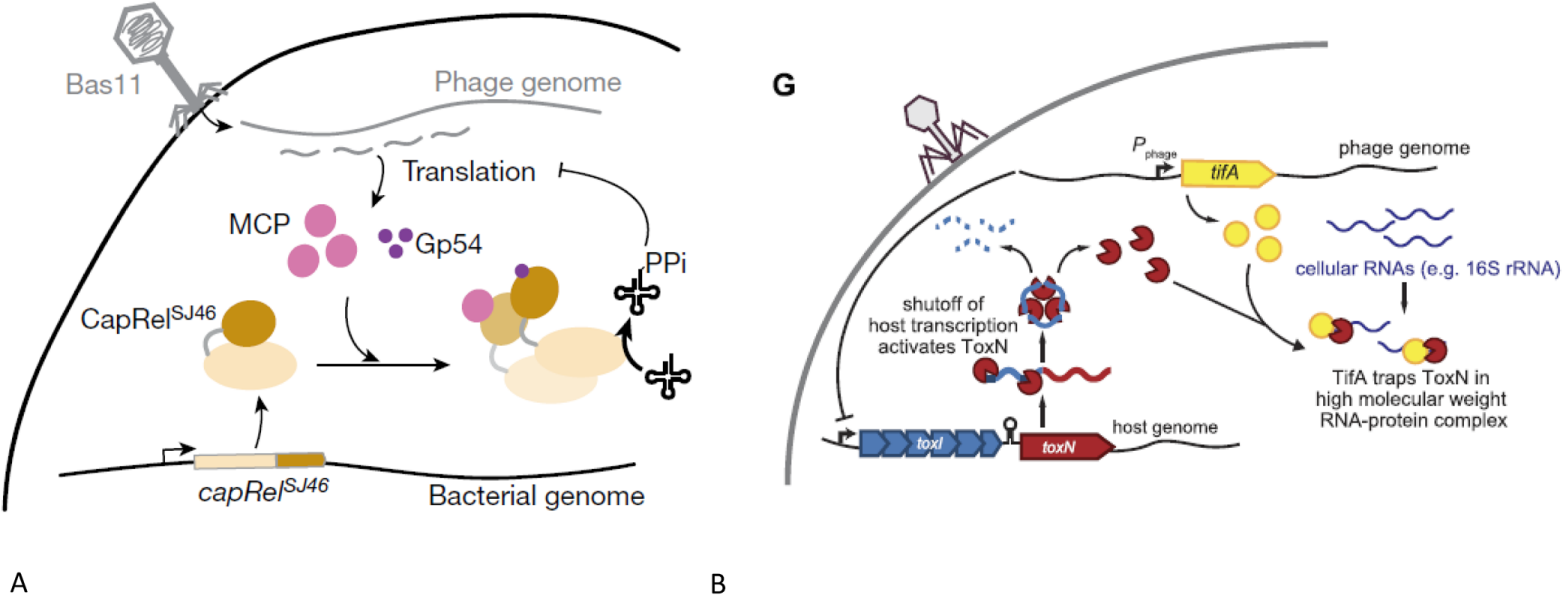
Toxin‐antitoxin systems as APS. (A) How sensing multiple phage proteins by the antitoxin renders phage escape from a toxin‐antitoxin APS difficult. The fused toxin‐antitoxin protein CapRelSJ46 dimerizes and the two antitoxin domains bind two distinct phage proteins (MCP, Gp54) liberating the toxin activity which pyrophosphorylates tRNA, blocking translation. Figure credit: Figures from Zhang et al. (2024), open access under a Creative Commons Attribution Licence 4.0. (B) How the phage T4 protein TifA inactivates the type III toxin–antitoxin system ToxIN. The antitoxin toxI encodes an array of short tandem RNA repeats to which the ToxN toxin binds. Phage infection leads to a shutoff of host transcription, including that of *toxI*, which liberates the ToxN RNase cleaving activity. The phage TifA protein traps ToxN in complexes with ribosomal RNA thereby preventing cleavage of phage transcripts. Figure credit: Figures from Guegler et al. 2024, open access under a Creative Commons Attribution Licence 4.0.

## 13 Escaping Detection by APS Sensors

Richmond‐Buccola et al. (2024) conducted a screen of 975 pairs of phages challenging Type I CBASS APS. CBASS APS comprised clades A–G of CD‐NTase enzymes that sense phage infections and synthesize eight different cyclic nucleotide based signalling molecules that activated diverse effector proteins, thus restricting phage replication. The tested phages were from the BASEL collection and represented diverse groups of phages. This screen of CBASS‐phage combinations were used to map host‐virus specificity of Type I CBASS immunity. Some Type I CBASS operons exhibit a broad range of defence against dsDNA phages from diverse families. In contrast, some phages particularly T4 were exceptional resistant to Type I CBASS. The researchers noted that mutations in virion assembly proteins, namely the prohead protease of T1 phages, enabled escape from Type I CBASS immunity. Viral structural proteins might thus serve as molecular cues for CD‐NTase to detect phage infection. The scientists demonstrated that the viral structural protein associates with the CD‐NTase. Based on these observations, it can be understood how mutations in a capsid scaffold protein or a major capsid protein of phages leads to immune evasion.

Nagy et al. (2026) screened phage proteins that might serve as triggers for a phage defence system. They used a library of plasmids expressing every open reading frame of six coliphages against diverse APS in a large number of *E. coli* cells. With this approach they identified 100 phage ORF‐ *E. coli* strain pairs that may represent novel phage stimuli. Activators of APS were diverse phage tail fibre proteins, their common denominator was that all required a phage chaperone for proper assembly. Likewise, the major phage capsid protein from several phages, sharing only low aa sequence identity, triggered APS. Here the only common denominator was their need for oligomerization.

When working with Thoeris APS in *Staphylococcus aureus* , Roberts et al. (2025) observed that the major head proteins (Mhp) of several staphylococcal and streptococcal phages are the necessary and sufficient trigger for the Thoeris APS action (NAD^+^ degradation, leading to abortive infection). In comparison with other Thoeris APS, the *S. aureus* system consists of two ThsB proteins instead of one ThsB protein. In uninfected hosts, ThsB1 and ThsB2 do not interact with each other. The researchers demonstrated experimentally that ThsB1 interacts physically with Mhp and that this complex recruits and activates ThsB2. Once activated, ThsB2 synthesizes the signalling molecule, which then triggers the effector function in ThsA. The scientists argued that the detection of the Mhp as trigger of Thoeris APS assures broad activity against many viruses, even including streptococcal phages due to sequence conservation. The important structural constraints on Mhp for phage virion formation assure that mutations are not easily obtained. Nevertheless, the researchers detected escape mutants that showed a single missense mutation in the Mhp of staphylococcal phages. These mutant phages failed to trigger the Thoeris APS and replicated efficiently. They suspected that this APS‐phage conflict caused the serological differentiation of staphylococcal phages caused by Mhp variation imposed by APS selection.

## 14 Arms Race Between Bacterial Defence and Bacteriophage Anti‐Defence Systems

Treating bacterial anti‐phage defence systems and phage countermeasures inactivating these systems separately as done in the present and previous editorial review is an artificial division of a single phenomenon. In fact, bacteria and bacteriophages are interlocked in an age‐old arms race where each side develops strategies to fight off the other side to which the adversary must find an answer in order to stay on the scene (Hampton et al. 2020). Aspects of this arms race can be read from a number of research reports. Perhaps the most obvious evidence for this arms race is provided by the wide distribution of defence and anti‐defence systems in bacteria and their corresponding phages. I will illustrate this aspect with *Pseudomonas aeruginosa* (PA), a widespread bacterium found in water, soil and plants which also represents an important human pathogen, particularly in immunocompromised patients.

### 14.1 Defence and Anti‐Defence in *P. aeruginosa* and Its Phages

Using the DefenseFinder bioinformatic program, the number of APS varied greatly from one to seventeen between PA strains. Even when comparing closely related PA strains, very different antiviral arsenals were detected (e.g., PA strain Ocean‐1155: 7 APS including RM, Abi2, AbiEii, CBASS, Gabija defences; vs. PA strain C79: 12 APS comprising RM, PrrC, Abi2, BREX, Gabija, CBASS, Shedu, and Gao defences). To explain their observation, researchers suggested APS acquisition by horizontal gene transfer under phage selection pressure depending on ecological conditions. Overall, the most common systems are RM and CRISPR‐Cas, followed by CBASS and Gabija, which were present in more than 40% of the strains (Tesson et al. 2022). Overall, PA strains carry about 70% of all currently known defence systems.

Costa et al. (2024) conducted an experimental APS search using 924 phage‐host combinations (28 phages against 33 hosts, including 32 antibiotic‐resistant clinical PA strains and the well‐studied PAO1 strain). Five clinical strains displayed 13 to 19 APS and were resistant to all 28 tested phages. Half of the strains contained adaptive immunity via CRISPR‐Cas APS; most of them were directed against temperate phages, which were not included in the virulent test phage panel. Fourteen of the identified non‐adaptive APS from the clinical isolates were experimentally expressed in the reference PAO1 strain and 9 inhibited phages from the test panel. APS Zorya, RADAR and CBASS conferred broad protection, indicating defence systems that use sensing and targeting mechanisms that rely on phage features shared among different phage families. The researchers found a strong correlation between the number of defence systems and phage resistance phenotype, suggesting that phage host range (which has traditionally been linked to receptor‐associated factors) will also strongly be influenced by the presence of APS. Defence systems, which exhibit complementary anti‐phage specificities, did co‐occur, indicating selection from successive phage infections.

In a complementary screen, Costa et al. (2025) searched APS defence genes in the pangenome of 162 PA phages belonging to the *Pbunavirus* phage group. They distinguished core genes (present in 95% of the phages), shell genes and cloud genes present in more or less than 1.5% of the phages, respectively. They identified a highly variable 15 kb region in *Pbunavirus*, mainly composed of small hypothetical proteins. Then they cloned 43 genes from this region and tested them individually in PAO1 against 12 APS. The researchers detected five anti‐defence genes targeting diverse APS. Two genes inactivated multiple defence systems and were widespread among phages infecting 17 bacterial genera. Others were specific for APS Zorya, Thoeris and Druantia. On average, phages of the *Pbunavirus* group encoded three anti‐defence genes per phage. No phage encoded all five anti‐defence genes, suggesting that the pangenome acts as a reservoir from which individual phages selectively acquire anti‐defence genes by recombination between phages. However, they failed to detect *Pbunavirus* anti‐defence genes in other genera of *Pseudomonas* phages, suggesting very little exchange of these genes between phage groups. None of these *Pbunavirus* anti‐defence genes shared similarity with previously characterized proteins, indicating that many such phage anti‐defence genes remain to be discovered.

### 14.2 Horizontal Gene Transfer: Apyc1

There are further indications for an intensive arms race between bacteriophages and bacteria. Some data suggest surprising relations between bacterial defence and phage anti‐defence systems. An illustration is phage‐encoded Apyc1 that cleave cyclic pyrimidines used as signalling molecules in the Pycsar APS (Hobbs et al. 2022). When developing their AntiDefenseFinder tool, Tesson et al. (2025) identified 1800 cases of Apyc1 encoded in bacterial chromosomes outside of mobile DNA elements. Upon phylogenetic analysis, phage‐derived Apyc1 are branching from bacterial Apyc1, suggesting bacterial Apyc1 as ancestral forms. Apparently, phages have acquired anti‐defence functions from bacterial genes that play regulatory functions in phage defence.

### 14.3 Horizontal Gene Transfer: CRISPR‐Cas

Another even more striking case is provided by the discovery of CRISPR‐Cas systems, the paradigm of the bacterial APS, that were encoded in phage genomes. Al‐Shayeb et al. (2022) discovered in huge metagenome screens 6000 CRISPR‐encoding phages. Compared to 40% of bacteria and 80% of archaea, only 0.4% of phages encode CRISPR‐Cas systems. Less than 10% of the phage‐encoded CRISPR‐Cas contained the machinery for the acquisition of new spacer sequences. All six known types of CRISPR‐Cas systems occurred in phages. Type III systems are associated with CRISPR arrays targeting vital or highly abundant RNA transcripts of other mobile elements, such as phage tail proteins or transposases. Apparently, phages have acquired bacterial anti‐phage systems that were repurposed to defend phages against the competition with other phages or mobile DNA elements. The arms race between bacteria and phages can thus in exceptional cases transit into co‐evolution if not cooperation. The latter case is illustrated by a phage‐encoded Type V CRISPR‐Cas12 system where the phage‐associated nuclease Cas12p forms complexes with the bacterial thioredoxin protein TrxA to enable target DNA degradation (Wang et al. 2026). In that specific case, a bacteriophage apparently co‐opts a bacterial factor to augment its own genome degradation machinery, potentially directed against competing phages.

### 14.4 Sensing Multiple Phage Proteins

Individual APS were anticipated to recognize only a single ligand during phage infection, suggesting a one‐to‐one relationship between bacterial sensor and phage trigger. However, when studying the APS protein CapRel^SJ46^, Zhang et al. (2024) observed a different situation. CapRel^SJ46^APS represents a fused toxin‐antitoxin protein (Figure 7A). The N‐terminal domain is the toxin; when activated, it pyrophosphorylates the 3′ end of transfer RNAs, which in turn inhibits protein translation and restricts phage propagation. The C‐terminal domain of CapRel^SJ46^ is the antitoxin. Binding of the major capsid protein (MCP) from phage SECф27 induces activation of the effector domain. A point mutation in MCP led to escape of phage SECф27 from CapRel^SJ46^control. Intriguingly, escape mutants from the closely related phage Bas11 mapped to Gp54, a small phage protein of unknown function. Escape by Bas11 Gp54 mutants was only partial; only when combined with MCP mutants complete escape of Bas11 from CapRel^SJ46^APS control was observed. The researchers demonstrated that the C‐terminal antitoxin domain binds and senses two completely unrelated and structurally different phage proteins. The two phage proteins bind to distinct but overlapping regions of antitoxin. This flexibility allows for more efficient phage detection and at the same time renders it more difficult for the phage to escape from defence, since two mutations in two different genes are needed.

### 14.5 Structure and Not Sequence Dependent Phage Protein Sensing

Proteins of the STAND superfamily show a tripartite structure consisting of a central NTPase domain, a C‐terminal sensor and an N‐terminal effector. Antiviral STANDs (Avs) provide protection against phage infections. By proteomic profiling with tagged Avs7, Lee et al. (2026) identified in phage T1 infected cells a complex formed by Avs7, the MCP of phage T1 and host translation elongation factor Tu. The complex degraded dsDNA. The Avs7 sensor domain adopted a bilobed structure that envelops the MCP. The host Tu factor binds to the outer surface of the flexible sensor region and enhances the effector nuclease activity. The researchers then cloned 30 Avs proteins and tested them for binding against a library of 600 genes from 36 mostly coliphages. The bound phage proteins encompassed most of the core structural and replicative proteins of tailed phages (Figure 8B). The list included the portal, portal adaptor, tail nozzle, head–tail connector, tail terminator, tail tube protein, tail assembly chaperone, tape measure protein, DNA polymerase, helicase and single‐stranded DNA annealing protein. Interestingly, Avs proteins recognized homologous proteins of different phages that shared little sequence similarity but displayed a comparable core folding. There was, however, minimal cross‐reactivity among non‐homologous phage proteins (Figure 8A). This antiviral strategy recognizing flexibly a single phage protein avoids autoimmunity against bacterial proteins. By recognizing phage proteins by their structural fold and not by a specific amino acid sequence, the individual Avs cover directly an entire group of related phages. The multitude of phage proteins sensed by a large set (> 30) of different Avs represents barriers to phages trying to escape from control by mutating a single protein.

**FIGURE 8:**
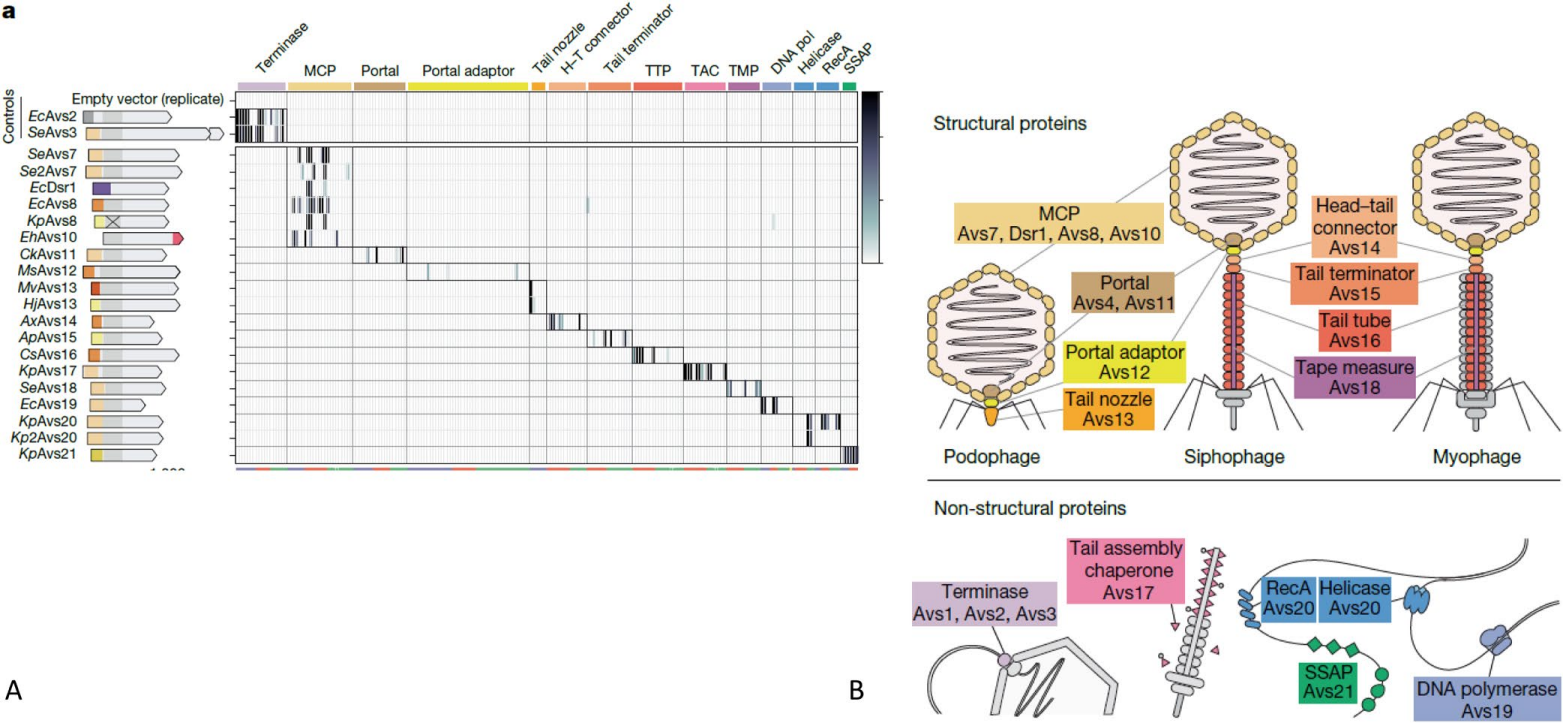
Diversity and specificity of phage protein sensing by a set of Antiviral STAND superfamily (Avs) proteins. (A) The heatmap displays the sensing of phage protein specified on the horizontal line by a diverse set of Antiviral STAND superfamily (Avs) proteins specified on the vertical line. With the exception of Avs20, each Avs recognizes a single phage protein. (B) The phage proteins recognized by the different Avs cover a broad range of structural and functional proteins. The location of structural phage proteins recognized by Avs is depicted on the phage particle and for the non‐structural proteins with their functional role in phage replication and maturation. Figure credit: Figure and legend from Lee et al. (2026), open access under a Creative Commons Attribution Licence 4.0.

### 14.6 The Stringent Response

Bacteria have developed an even more versatile strategy to impair phage replication: the stringent response (SR). This global stress survival mechanism system recognizes nutrient depletion or general physiological stress and focuses the cell on survival instead of growth. The SR activates the RelA and SpoT enzymes synthesizing the alarmone nucleotide (p)ppGpp. The alarmone inactivates the host RNA polymerase and thus slows down the cell metabolism. Initially, SR favours the take‐over of the cell by phage transcription. However, later in the replication cycle when the phage needs elevated protein synthesis and energy production by the cell, the SR impairs phage growth by most phages, irrespective of their nature. Phages have again found ways to impair the SR either by elaborating a phage‐encoded alarmone‐degrading enzyme ATD116, a pyrophosphohydrolase (Ho et al. 2023) or by the moonlighting activity of the portal protein of phage T7 appearing late in the infection process which engages RelA and SpoT enzymes preventing the sustained synthesis of the alarmone (Kronborg et al. 2026) (Figure 9A).

**FIGURE 9:**
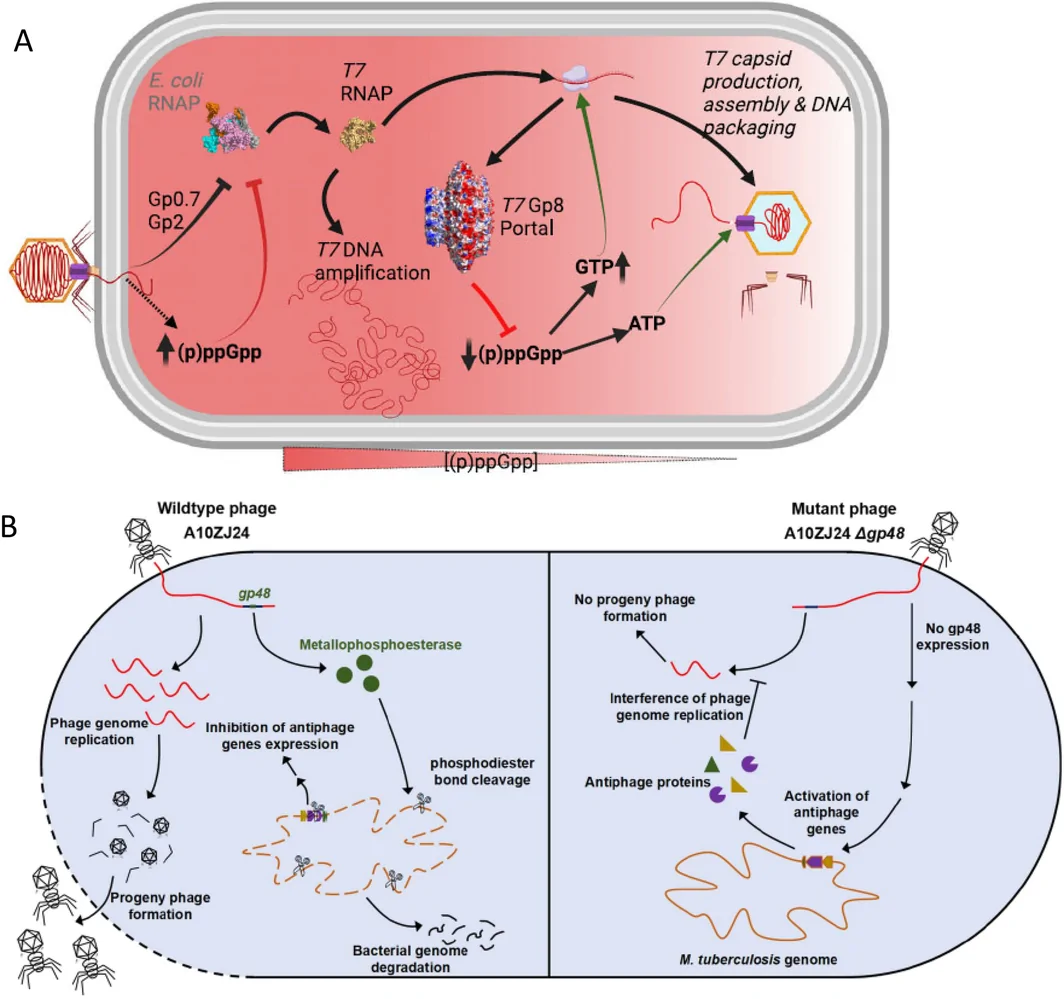
A generalized bacterial APS and a generalized phage countermeasure that do not depend on the specificity of bacterium‐phage interactions. (A) The stringent response is a global stress survival mechanism also active during phage infection. Bacteria elaborate a (p)ppGpp alarmone that suppresses host transcription which inhibits late phage maturation steps. The T7 phage portal protein Gp8 inhibits the alarmone synthetases RelA and SpoT which restores the cell’s synthetic capacity to allow phage maturation. Figure credit: Figure and legend from Kronborg et al. (2026), open access under a Creative Commons Attribution Licence 4.0. (B) A general phage countermeasure against APS consists of a rapid degradation of the host genome after infection such that the bacterial APS cannot be expressed. This strategy is here illustrated for mycobacteriophage A10ZJ24. Phage lacking gp48, a metallo‐phosphoesterase that degrades the bacterial genome, is suppressed by the expression of the cell’s APS. Figure credit: Figure and legend from Li et al. (2025), open access under a Creative Commons Attribution Licence 4.0.

### 14.7 Bacterial Genome Degradation

As bacteria use SR as an anti‐viral system that does not depend on the specific recognition of phages, phages have evolved anti‐defence strategies that undermine APS without the need to recognize specific anti‐viral system. This strategy is illustrated by the mycobacteriophage‐encoded Gp48, a metallo‐phosphoesterase, which is expressed early during phage infection and disrupts mycobacterial genomic DNA integrity, thereby silencing the expression of multiple anti‐phage defence genes. In this way, the antidefense strategy does not depend on the specific recognition of APS to inactivate them (Li et al. 2025) (Figure 9B). Bacterial host genome degradation early after infection is a widespread phage strategy, for example, phage T4. This approach does not only inactivate genes executing anti‐phage functions but also provides nucleotide precursors for phage genome replication. To counter this strategy of phage T4, bacteria have evolved the Metis APS to which phage T4 reacts with mutating the endonuclease responsible for host genome degradation (see above).

## 15 Outlook

We live currently through a renaissance of phage research. The deciphering of the molecular details for the arms race between bacteria and bacteriophages has opened new vistas for molecular biology. Despite decades in phage research the current analysis of bacterial anti‐phage functions and phage anti‐defence mechanisms has widened our understanding of phage biology. The sheer amount of T4 phage genes of unknown function in one of biology's best investigated systems is a reminder how much still remains to be learned from phage research. Beyond fundamental molecular biology insights, research on the arms race between bacteria and its viruses has implications for many fields ranging from genetic engineering tools for molecular biotechnology (Trost et al. 2024; Jia and Patel 2021) to evolutionary insights on ancient links between antiviral immunity in bacteria and eukaryotes (Kranzusch 2026) and the cost and regulation of anti‐viral defence (Shu et al. 2026). Phage‐bacterium interactions influence the evolution of important human pathogens (Barton et al. 2026; Mathur et al. 2026) and the ecology of marine bacteria (Nadel et al. 2025) with possible impact on climate. More down to practical problems, this arms race has biotechnology implications for the development of phage‐resistant bacterial starter strains for the food industry as well as the development of therapeutical phages against antibiotic‐resistant bacterial pathogens.

## Author Contributions

**Harald Brüssow:** conceptualization, investigation, writing – original draft, writing – review and editing.

## Funding

The author has nothing to report.

## Disclosure

Artificial intelligence tools were not used in the preparation of this editorial review.

## Conflicts of Interest

The author declares no conflicts of interest.

## Supporting information


**Figure S1:** Illustration how phages overcome bacterial CRISPR‐Cas systems. Left side: CRISPR‐Cas systems are adaptive defence systems that insert nucleic acid fragments from previous infections into the CRISPR array. Sequences from the CRISPR array are transcribed into CRISPR RNAs (crRNAs) that recognize complementary nucleic acids. crRNA binds Cas protein(s), forming the effector complex that recognizes and cleaves the complementary nucleic acid, preventing phage proliferation. Additionally, the type III CRISPR‐Cas system synthesizes cyclic oligoadenylate (cOA), which induces non‐targeted nucleic acid cleavage. Right side: Phages have developed a variety of functionally distinct anti‐CRISPR proteins (acr) to counteract defence systems. AcrIIC3 prevents effector complex assembly by causing dimerization of Cas9; AcrVIA1 targets RNA recognition by binding to the effector complex; AcrIF3 inhibits DNA cleavage by binding to the nuclease Cas3 and obstructing its interaction with the effector complex; AcrIII‐1 inhibits non‐targeted RNA cleavage by degrading cOA utilized by the type III CRISPR‐Cas system. Figure credit: Figure and legend from Murtazalieva et al. (2024), open access under a Creative Commons Attribution License 4.0.
**Figure S2:** Illustration how phages overcome the bacterial defence systems Thoeris, Pycsar, and CBASS. Left side: Upon detecting phage infection, these APS synthesize cyclic nucleotides. Specifically, Thoeris APS produces 1″–2′ glycocyclic adenosine 5′‐diphosphate ribose (gcADPR); the Pycsar system produces cyclic CMP or UMP; and the CBASS system synthesizes cyclic di‐ and trinucleotide (CDN and CTN) signalling molecules. These signalling molecules activate downstream effectors, leading to cell death frequently via depletion of NAD+. Right side: Phages have developed different strategies to evade these defences. Tad1, Tad2, and Acb2 proteins sequester signalling molecules thus preventing the activation of the downstream effectors whereas Acb1 and Apyc1 degrade the signalling molecules; and some phages encode NAD+ reconstitution pathways that restore cellular NAD+. NMPs, nucledotidemonophospates. Figure credit: Figure and legend from Murtazalieva et al. (2024), open access under a Creative Commons Attribution License 4.0.

## Data Availability

Data sharing not applicable to this article as no datasets were generated or analysed during the current study.
