# Supplementary material for: The Arms Race Between Bacteria and Their Viruses: Update on Phage Countermeasures Against Bacterial Defence Systems
Source: Microb Biotechnol. 2026 Nov 2;19(11):e70462. doi: 10.1111/1751-7915.70462 (PMC13728456; doi:10.1111/1751-7915.70462)
Supplement: Supplementary file 1 [file MBT2-19-e70462-s001.docx]

Figure.S1 Illustration how phages overcome bacterial CRISPR-Cas systems. Left side: CRISPR-Cas systems are adaptive defence systems that insert nucleic acid fragments from previous infections into the CRISPR array. Sequences from the CRISPR array are transcribed into CRISPR RNAs (crRNAs) that recognize complementary nucleic acids. crRNA binds Cas protein(s), forming the effector complex that recognizes and cleaves the complementary nucleic acid, preventing phage proliferation. Additionally, the type III CRISPR-Cas system synthesizes cyclic oligoadenylate (cOA), which induces nontargeted nucleic acid cleavageRight side: Phages have developed a variety of functionally distinct anti-CRISPR proteins (acr) to counteract defence systems. AcrIIC3 prevents effector complex assembly by causing dimerization of Cas9; AcrVIA1 targets RNA recognition by binding to the effector complex; AcrIF3 inhibits DNA cleavage by binding to the nuclease Cas3 and obstructing its interaction with the effector complex; AcrIII-1 inhibits nontargeted RNA cleavage by degrading cOA utilized by the type III CRISPR-Cas system. Figure credit: Figure and legend from Murtazalieva et al. (2024), open access under a Creative Commons Attribution License 4.0.

Figure S2: Illustration how phages overcome the bacterial defense systems Thoeris, Pycsar, and CBASS. Left side: Upon detecting phage infection, these APS synthesize cyclic nucleotides. Specifically, Thoeris APS produces 1’’–2’ glycocyclic adenosine 5’-diphosphate ribose (gcADPR); the Pycsar system produces cyclic CMP or UMP; and the CBASS system synthesizes cyclic di- and trinucleotide (CDN and CTN) signaling molecules. These signaling molecules activate downstream effectors, leading to cell death frequently via depletion of NAD+. Right side: Phages have developed different strategies to evade these defenses. Tad1, Tad2, and Acb2 proteins sequester signaling molecules thus preventing the activation of the downstream effectors while Acb1 and Apyc1 degrade the signaling molecules; and some phages encode NAD+ reconstitution pathways that restore cellular NAD+. Abbreviation: NMPs, nucledotidemonophospates. Figure credit: Figure and legend from Murtazalieva et al. (2024), open access under a Creative Commons Attribution License 4.0.
